# Changes in Food Intake in Australia: Comparing the 1995 and 2011 National Nutrition Survey Results Disaggregated into Basic Foods

**DOI:** 10.3390/foods5020040

**Published:** 2016-05-25

**Authors:** Bradley Ridoutt, Danielle Baird, Kathryn Bastiaans, Gilly Hendrie, Malcolm Riley, Peerasak Sanguansri, Julie Syrette, Manny Noakes

**Affiliations:** 1 Commonwealth Scientific and Industrial Research Organisation (CSIRO) Agriculture, Private Bag 10, Clayton South, Victoria 3169, Australia; 2 Department of Agricultural Economics, University of the Free State, Bloemfontein 9300, South Africa; 3 CSIRO Food and Nutrition, PO Box 10041, Adelaide, South Australia 5000, Australia; Danielle.Baird@csiro.au (D.B.); Kathryn.Bastiaans@csiro.au (K.B.); Gilly.Hendrie@csiro.au (G.H.); Malcolm.Riley@csiro.au (M.R.); Julie.Syrette@csiro.au (J.S.); Manny.Noakes@csiro.au (M.N.); 4 CSIRO Food and Nutrition, Private Bag 16, Werribee, Victoria 3030, Australia; Peerasak.Sanguansri.csiro.au

**Keywords:** Australian Guide to Healthy Eating, Australian Health Survey, dietary transition, eating habits, food consumption pattern, food choices, food intake, National Nutrition and Physical Activity Survey

## Abstract

As nations seek to address obesity and diet-related chronic disease, understanding shifts in food intake over time is an imperative. However, quantifying intake of basic foods is not straightforward because of the diversity of raw and cooked wholefoods, processed foods and mixed dishes actually consumed. In this study, data from the Australian national nutrition surveys of 1995 and 2011, each involving more than 12,000 individuals and covering more than 4500 separate foods, were coherently disaggregated into basic foods, with cooking and processing factors applied where necessary. Although Australians are generally not eating in a manner consistent with national dietary guidelines, there have been several positive changes. Australians are eating more whole fruit, a greater diversity of vegetables, more beans, peas and pulses, less refined sugar, and they have increased their preference for brown and wholegrain cereals. Adult Australians have also increased their intake of nuts and seeds. Fruit juice consumption markedly declined, especially for younger Australians. Cocoa consumption increased and shifts in dairy product intake were mixed, reflecting one of several important differences between age and gender cohorts. This study sets the context for more detailed research at the level of specific foods to understand individual and household differences.

## 1. Introduction

Much has been written about dietary change, especially in relation to developing countries and economies in transition where there is rapidly increasing adoption of so-called Western diets [1,2,3]. Such diets, when characterized by high levels of saturated fat, sugar and refined foods, and low levels of fiber, are associated with alarmingly increased rates of overweight and obesity and a variety of chronic diseases [4,5,6]. Dietary change is also of importance from the perspective of environmental sustainability and there is a steadily growing interest in the subject of sustainable diets [7,8,9]. The food system has a critical relationship to several of the planetary boundaries [10], with greenhouse gas emissions, impacts related to land and water use, and diffuse nutrient emissions of particular importance [11,12,13]. In many cases, a shift from a traditional to a Western diet is also associated with an increased consumption of livestock products and this has raised additional concerns from the perspective of environmental sustainability [14]. Dietary transitions in developed countries, though usually much more subtle in comparison, are also of acute interest for essentially the same reasons.

In Australia, overweight and obesity are regarded as major health and societal issues [15]. According to the most recent Australian Health Survey, conducted in 2011–2012, the proportion of overweight and obese children has increased to almost 26% [16]. At the same time, almost 56% of adult women and almost 70% of adult men were overweight or obese [16]. Not only is the overall prevalence of overweight and obesity increasing, but the balance is also shifting from overweight towards obesity [17]. Dietary risks, along with high body mass index, are the leading risk factors contributing to the burden of disease in Australia [18]. The total cost of obesity, including both direct costs to the healthcare system and indirect costs associated with lost productivity, lost wellbeing and carer costs, has been estimated at more than AUD $58 billion per annum [15]. From the perspective of food production, though Australia is a net food exporter, concern is also regularly expressed about future food security, especially in the context of climate change and water scarcity [19,20,21,22,23,24,25,26,27,28,29]. Changing patterns of food consumption are therefore important for a variety of reasons and to a wide range of stakeholders.

However, dietary changes are not easily quantified. Economists refer to apparent consumption, being the sum of domestic production plus imports minus exports, sometimes also adjusted for changes in stocks. This can provide an indication of the total quantity of food in a national food system. However, this does not represent the quantity of food actually eaten as 30% of food or more can be wasted and the wasted proportion is not consistent across different food products [30,31]. Furthermore, the total food supply does not inform about variation in food intake by age and gender and other socio-economic factors.

National dietary surveys are another source of valuable information about dietary patterns. However, these surveys are primarily designed to assess nutrient intake and cannot readily be used to assess variation in intake of particular foods. This is because participants report intake of a wide range of wholefoods, processed foods and mixed dishes. For example, a participant might report the addition of sugar to tea or coffee. However, their main intake of sugar could be through the consumption of processed foods with added sugar. Similarly, meat might be consumed as a specific food item, but it might also be included as a minor ingredient in a sandwich, or as a pizza topping. Eggs and milk can be eaten as separate foods, but they are also very common food ingredients. The manner in which dietary intake data are coded generally involves classifying mixed dishes according to their primary ingredient by mass. A meat pie or a hamburger might therefore be categorized as cereal-based products.

The purpose of this study was to quantify change in intake of basic foods in Australia according to gender and age group. By basic foods we refer to different types of fruit, vegetables, meat, eggs, nuts, *etc*. This involved complete disaggregation of food intake data from the two most recent national nutrition surveys covering the periods 1995–1996 [32] and 2011–2012 [33]. Similar activities have been undertaken in the past, but limited in scope to specific foods, such as beef, lamb and veal [34] and dairy foods [35] and to specific time periods. Disaggregation is becoming increasingly important to understand actual food intake because Australians are consuming fewer meals prepared at home from basic food ingredients, as meals are increasingly purchased or prepared from ready-to-eat or semi-prepared components [36]. As such, this study offers a unique perspective on dietary change in Australia intended to be of interest to public health nutrition professions and a broad range of individuals and organizations concerned about Australia’s food future. It forms part of a wider study of the trajectories of food demand and production in Australia to 2050 [37].

## 2. Materials and Methods

### 2.1. Food Intake Data

Reported food intake data were obtained from the two most recent national nutrition surveys conducted in Australia: The National Nutrition Survey of 1995 [32] and the Australian Health Survey of 2011 [33]. These are both large national surveys of dietary intake, each involving more than 12,000 participants and covering all age groups (2 years and above) and with nationally representative coverage of urban and rural communities. Each survey was conducted over 13 months, in order to capture variation in eating patterns according to season, and included all days of the week. Originally adapted from the interview procedure developed by the USDA for the Continuing Survey of Food Intakes by Individuals, the primary method of data collection was a highly detailed quantitative survey where participants recall and describe all food and beverages and portion sizes consumed (*i.e.*, eaten) during the previous 24-h period (midnight to midnight). In each case, more than 4500 individual foods were reported to have been eaten.

While the 1995 and 2011 surveys were broadly the same, some differences also occurred. For instance, the 1995 survey was conducted between February and March the following year, whereas the 2011 survey was conducted between May and June the following year. There were also minor differences in the proportion of sampling by season and by day of the week. In addition, there were differences in response rate (61% in 1995, 77% in 2011) and in the practical administration of the survey. In 1995, the survey was conducted by nutritionists trained as interviewers. In contrast, professional interviewers conducted the survey in 2011 and computers were introduced into the interview process [38]. That said, both surveys were intended to achieve a generalizable population estimate of nutrient intake delineated by age, gender and geographical groupings.

### 2.2. Disaggregation into Basic Foods

Disaggregation of the food intake data was undertaken systematically by a team of five individuals with expert knowledge of food recipes and composition. The task was straightforward in some cases (e.g., a raw apple), but complex in others (e.g., a chocolate chip biscuit consisting of wheat, sugar, shortening, cocoa, egg and milk as components). In the absence of specific information about mixed dishes, archetypal recipes were applied. For example, apple crumble desserts were coded as cooked apple (73%), refined white wheat (6%), refined sugar (5%), butter (7%) and water (9%). Some foods (e.g., Pavlova dessert) include only part of a basic food (e.g., egg white only). Care was taken to avoid double counting (e.g., whole eggs, egg whites, egg yolks). Basic food categories included 28 fruits, 21 vegetables, mushrooms and truffles, 8 types of meat and seafood, 7 types of grain, eggs, 5 types of dairy products, dried beans, peas and pulses, nuts, honey, refined sugar, *etc.* Foods were sometimes consumed raw and at other times cooked. Some foods increase in mass during preparation (e.g., rice), whereas others lose mass due to dehydration (e.g., pan fried meat). To facilitate analysis and reporting, cooked food portions were translated into raw quantities using conversion factors predominantly taken from the NUTTAB reference database [39]. In addition, conversion factors were used to translate processed foods into basic food quantities. For example a quantity of dried fruit requires 2.9 to 4.0 times as much fruit, depending on the type of fruit used. These processing conversion factors were obtained from a variety of sources [39,40,41]. The application of cooking and processing factors enabled the results to be expressed as raw equivalents (edible portion only).

### 2.3. Adjustment for Under-Reporting

The major limitation of the nutrition survey method, with relevance to this study, relates to the 24-h recall approach and the possibility that people did not always recall accurately the foods and portion sizes eaten. There is also the possibility of deliberate misreporting. In order to assist in the interpretation of the nutrition survey data, the Australian Bureau of Statistics analyzed the potential under-reporting prevalence and estimated how much energy might be missing from the food recall data [38]. For the purpose of this study, these estimates of food energy under-reporting were applied uniformly across all foods. The scaling factors were 16% and 1% for females and males in 1995, and 21% and 17% for females and males in 2011. Whilst it is possible that under-reporting was biased toward certain types of foods (e.g., discretionary foods), insufficient evidence existed to support a method which involved allocation of under-reported food energy to specific foods.

### 2.4. Population Age Class Distribution for Reporting

By applying the same survey weights as the Australian Bureau of Statistics, the 1995 and 2011 national intake of basic foods was estimated (by age and gender cohort). In order to report overall changes in food intake in Australia, the data for all adults (19 years and above) and all children (2 to 18 years) were aggregated based on the 2011 age class distribution.

## 3. Results

### 3.1. Food Choices of Adult Australians

In comparing disaggregated food intake data from 1995 and 2011, important shifts in food choice in Australia become apparent. Overall, it can be seen that on a per capita basis adult Australians have substantially increased their intake of wine (+44%), cocoa (+84%), nuts and seeds (+61%), beans, peas and pulses (+48%), seafood (+37%) and edible seaweed (>400%, albeit from a very low base, *i.e.*, <0.01 kg/person/year in 1995) (Table 1). The largest per capita decreases in intake were for beer (−9%), butter and ghee (−9%) and vegetable oil (−11%). Only minor change was observed for fruits and vegetables, grains and sugar. That said, the form of consumption shifted in important ways for several food groups (Table 2, Table 3, Table 4, Table 5 and Table 6). Although per capita adult fruit intake (in all forms) declined by a modest 0.9% (Table 1), raw fruit intake increased by 13.6%, whereas intake of fruit in the form of juice decreased by 8.5% (Table 2). Overall, per capita adult vegetable intake decreased by 2.7% (Table 1). However, this was the combination of a decrease in intake of potato and other starchy roots (−28%), but an increase in intake of mushrooms (+16%) and other vegetables (+7%) (Table 3). While total per capita adult intake of meat increased (Table 1), intake of beef and lamb decreased (−2.6% and −7.7%, respectively), whereas intake of all other meats, including seafood, increased. Adult intake of chicken increased by almost 50% from 19.3 to 28.9 kg/person/year (Table 4). The largest proportional increase in adult meat intake was for duck (*i.e.*, +450%, from a low base of 0.1 kg/person/year in 1995). The major shift in per capita adult intake of dairy products was away from whole milk and toward reduced fat milk and other further transformed dairy products like cheese, cream and yoghurt (Table 5). For grains, a shift toward rice was evident (Table 6). Intake of oats also increased substantially (+38%). The decrease in per capita adult intake of wheat was mainly associated with a decrease in intake of products from refined (white) wheat (−4.5%, Table 6). Patterns of change in per capita food intake were not always consistent across age and gender cohorts (Tables S1 and S2). For example, the substantial increases in wine and cocoa intake were associated more with the cohorts aged 50–69 and 70+ years, rather than adults aged 19 to 49. Observed increases in dairy product intake were stronger for adult males than females. Decreases in adult fruit juice consumption were predominantly associated with women (Tables S1 and S2).

### 3.2. Food Choices of Younger Australians

For younger Australians (aged 2–18 years), many of the per capita changes in food intake from 1995 to 2011 mirrored the changes described above for adult Australians (Table 1). These changes included substantial increases in per capita intake of cocoa (+33%), beans, peas and pulses (+64%), meat (+14%) and edible seaweed (almost 3000%, albeit from a tiny base of <0.01 kg/person/year in 1995), as well as a decrease in intake of vegetable oil (−12%). In contrast to adults, per capita intake of nuts and seeds by younger Australians decreased (−11%) and the increase in per capita intake of seafood was modest (<1%). Other decreases included dairy products (−8%), fruit (−11%) and sugar (−23%). For other food groups the changes were not so marked (Table 1). In common with adults, interesting shifts in the form of intake were also observed for younger Australians. The change in form of fruit intake was even more pronounced than for adults, with per capita raw fruit intake by younger Australians increasing by almost 35% and intake of fruit in the form of juice decreasing by almost 30% (Table 2). Additionally, for vegetables, the per capita intake of potato and other starchy roots was down by more than 35%, whereas intake of mushrooms and other types of vegetables increased (+33% and +28% respectively, Table 3). Younger Australians increased their per capita intake of most forms of meat, the exceptions being lamb (−16%) and seafood other than fish (−32%, Table 4). Per capita intake of whole milk and reduced fat milk both declined for younger Australians, although the shift was greatest for whole milk (Table 5). In contrast to fresh milk, younger Australians increased per capita intake of other dairy products (cheese, cream and yogurt, Table 5). Unlike adult Australians, younger Australians increased their overall per capita intake of wheat, especially whole-meal (brown) wheat (Table 6) The large increase in per capita intake of white rice observed for adult Australians (+24%, Table 6) was not evident for younger Australians. Whereas adult intake of oats markedly increased (+38%, Table 6), for younger Australians the intake of oats actually declined. Patterns of change in per capita food intake also varied according to age and gender cohort (Tables S1 and S2). Considering vegetables other than potatoes and starchy roots, in every age cohort boys increased their per capita intake to a greater extent than girls. Large (20% to 50%) decreases in per capita fruit juice intake were observed for all age groups of boys and girls, with the exception of teenage boys where the per capita decreases in intake were much lower (1% to 5%; Tables S1 and S2).

## 4. Discussion

Over the course of the study period (1995–1996 to 2011–2012), Australians have been exposed to a wide range of campaigns seeking to encourage healthy eating and regular physical activity, as well as warn about the dangers of overweight and obesity. These campaigns have been run by Federal and State governments (e.g., [42,43]) as well as a variety of non-government, non-profit, community based organizations (e.g., [44,45]). At the national level, these campaigns have included Get Moving [46] which encouraged greater levels of physical activity among children, Go for 2&5 [47] which encouraged increased intake of fresh fruit and vegetables, Measure Up [48] which highlighted the link between increased waist measurement and the risk of chronic disease, Swap It Don’t Stop It [48] which encouraged small changes toward healthy eating and lifestyle, and more recently Shape Up [43]. A variety of strategies have been used to reach the Australian public, including prominent print and national television advertising campaigns (e.g., [49]). The CSIRO Total Wellbeing Diet [50] and related programs have also been prominent in Australian society. These initiatives have all emphasized eating habits which are consistent with national dietary guidelines described in the Australian Guide to Healthy Eating [51]. Generally, Australians need to reduce consumption of energy-dense and nutrient poor discretionary foods and eat more whole fruit, vegetables, legumes and dairy products or alternatives [52]. However, for the reasons mentioned in the Introduction, it is not straightforward to quantify the intake of different basic foods, which is needed in order to assess the trajectory of the national diet and the outcomes of efforts to promote healthy eating habits.

The changes in food intake from 1995 to 2011 that have been quantified in this study are positive in many ways, although not all. In regards to fruit, the Australian Dietary Guidelines [51] recommend a minimum of one serve per day for two to three years olds, increasing to a minimum of 1.5 serves per day for four to eight year olds and at least two serves per day for older children, adolescents and adults. In 2011, this equated to an average intake of at least 121.5 kg/person/year for adults and 106.9 kg/person/year for children (2 to 18 years combined). Australians consume this quantity of fruit, but only when all forms of fruit intake are counted, including fruit juice (Table 1 and Table 2). Compared to most countries, Australians are high consumers of fruit juice [53]. The Australian dietary guidelines recommend that fruit be consumed whole and that fruit juice should be consumed only occasionally and in small amounts [51]. In this regard it is positive that fruit juice intake has declined, especially for younger Australians, and that intake of whole fruit has increased. That said, whole fruit intake remains less than half the recommended minimum level for total fruit intake. These results are for adults and younger Australians in aggregate and do not portray potentially important differences at the individual and family levels [54,55].

In regard to vegetables, the Australian Dietary Guidelines [51] recommend a minimum of 2.5 serves per day for two to three year olds, increasing to a minimum of 5 to 5.5 serves per day for older children and adolescents and five to six serves per day for adults. In 2011, this equated to an average intake of at least 153.3 kg/person/year for adults and 132.9 kg/person/year for children (2 to 18 years combined). From 1995 to 2011, total intake of vegetables (including mushrooms) underwent little change and remained well below the recommended minimum levels (Table 1). That said, there was a shift away from potatoes and other starchy roots towards other types of vegetables (Table 3). There was also a large increase in the consumption of beans, peas and pulses (48% for adults and 64% for younger Australians, Table 1). This is positive as the Australian Dietary Guidelines emphasize consumption of vegetables of different types and colors, as well as legumes and beans. Nevertheless, progressing toward adequate vegetable consumption remains a public health nutrition challenge in Australia, as it is elsewhere [56]. In Australia, the vegetable production industry is growing at a slower rate than the growth in demand, meaning that a situation of net import dependence arose in the past decade and the gap is projected to increase over time [37]. This situation is unlikely to support efforts to increase vegetable intake in the community. One consequence could be a closer link between international commodity prices and local retail prices, which in the event of a price spike could result in a barrier to purchase by lower income households.

Concerning meat, the Australian Dietary Guidelines [51] consider lean meats and poultry, fish, eggs, tofu, nuts and seeds, and legumes/beans jointly. Depending on age, the Guidelines recommend between one and three serves of these foods each day, and 3.5 serves during pregnancy. In comparing food intake in 1995 and 2011, what is evident is that Australians are generally eating more diversely in this food group (Table 1). Adults are eating more nuts and seeds, beans/peas and pulses, as well as eggs. Adults are also eating a wider variety of meats (Table 4). This is generally also the case for younger Australians, but with some exceptions, such as nuts and seeds where intake has decreased in most age and gender cohorts (Tables S1 and S2). The Guidelines [51] do make a specific recommendation regarding red meat, which is to consume a maximum of 455 g lean, cooked, red meat per week. While there are different methods of cooking red meat, many will lead to desiccation during cooking. Using the same cooking factors applied in this study, 455 g of cooked red meat per week equates to 31.5 kg of raw red meat per year. Australian adult consumption of red meat (beef and lamb) has fallen from 38.6 kg/person/year in 1995 to 37.2 kg/person/year in 2011 (Table 4). On average, Australian adults have reduced their intake of red meat, but are still consuming more than the recommended maximum. However, on average, men consumed more red meat than women, meaning that it is mostly men who could benefit from further reductions in red meat intake. The Guidelines [51] suggest that some children and young women may actually benefit from an increase in red meat consumption. We found red meat intake by females aged 14 to 18 and 19 to 49 to average 26.5 kg/person/year and 27.1 kg/person/year respectively, which lends some support to this recommendation.

The Guidelines [51] also make a specific recommendation in regard to seafood, which is for older children and those adult Australians who consume foods from animal sources, to eat two serves of fish per week, and for younger children to consume up to around 1 to 1.5 serves per week. Using the same cooking factors applied in this study, this equates to an intake of 12.0 kg of raw fish per person per year for adults and 6.0 to 9.0 kg/person/year for children. Since 1995, adult Australians have increased their average intake of fish from levels well below the recommendation to meet the recommendation in 2011 (Table 4). However, this did not similarly occur for children.

As to dairy products, the Australian Dietary Guidelines [51] recommend the consumption of a wide variety of mostly reduced fat products or alternatives. The minimum levels of intake range from 1.5 to 4 serves per day, depending on age and gender, which equated to an intake of dairy products equivalent to 290.7 kg/person/year of fluid milk for adults and 267.3 kg/person/year of fluid milk for younger Australians (aged 2 to 18, based on 2011 age distribution). From 1995 to 2011, adult Australians increased their average dairy product intake, but not nearly enough to reach the minimum recommended level (Table 1). Other positives were the shift from full cream milk toward reduced fat milk and the increased intake of yogurt (Table 5). Somewhat concerning was the observed reductions in dairy product intake by younger Australians, meaning that the average level of intake was even further away from the recommended minimum in 2011 compared to 1995 (Table 1). The statement in the Australian Dietary Guidelines [51] that most Australians need to consume more reduced fat milk, yogurt and cheese should therefore be underscored, notwithstanding the option of non-dairy calcium providing alternatives.

Another important shift in Australian food intake was a modest increase in the proportion of brown or wholegrain cereals, which is consistent with Australian Dietary Guideline recommendations [51]. Australians are also consuming less added sugar and butter. The reduced intake of added sugar was observed across all age and gender cohorts for younger Australians. In part, this is explained by a decrease in per capita consumption of sugar sweetened beverages in Australia over time [57]. These results for sugar are consistent with other evidence that refined sugar consumption has generally decreased in Australia, the UK, the USA and Canada [58,59,60]. The average adult intake of added sugar in Australia (20.4 kg/person/year) was very similar to the average intake in the Netherlands (55.4 g/person/day or 20.2 kg/person/year; [61]). In contrast with the decrease in refined sugar intake, cocoa intake increased, by almost 32% for younger Australians and 84% for adults. The Australian Dietary Guidelines [51] note that most Australians need to eat less chocolate.

Our study has a number of limitations, in part related to the limitations of the 24-h dietary recall method used in the national dietary intake surveys, the possibility of errors in portion size estimation, and the difficulties adjusting for under-reporting. According to Australian Bureau of Statistics estimates, under-reporting has increased from 1995–1996 to 2011–2012. As such, scaling factors were applied to the dietary intake data to improve the comparability of the two datasets, as described in Section 2.3. The estimates of food intake are therefore marginally higher than may be found in other studies where adjustments for under-reporting are not applied. In addition, the use of alternative scaling factors would have a bearing on the absolute results. Nevertheless, we have confidence in the general approach. The results we report are consistent with a broad range of market survey and anecdotal evidence [36]. The Australian National Nutrition Survey of 1995 has already formed the basis of a very wide range of published nutritional studies, and the nutrition component of the 2011 Australian Health Study has recently been used to report on topics as diverse as micronutrient intake [62,63], eating occasions [64,65] and portion size [66]. In addition, the food intake data, when aggregated across the Australian population, result in quantities consumed, which are sensible relative to estimates of national food supply and food waste [37,67].

## 5. Conclusions

In summary, although Australians are generally not eating in a manner consistent with national dietary guidelines [51], there have been several positive changes from 1995–1996 to 2011–2012. For example, Australians are generally eating more whole fruits, a greater diversity of vegetables, more beans, peas and pulses, less refined sugar, and they have increased their preference for brown and wholegrain cereals. Adult Australians have also increased their intake of nuts and seeds. Although fruit juice consumption per capita has declined, especially for younger Australians, it is still generally consumed at levels considerably above what could be considered occasional. The substantial increase in chocolate consumption could also be viewed as a negative from a health perspective. These high-level shifts in food choice described in this study are likely to be of interest to public health nutrition professionals who have responsibility for the design and implementation of healthy eating campaigns, as well as practicing dietitians, and a broad range of stakeholders in the food industry. This study also sets the context for more detailed research at the level of specific foods to understand individual and household differences in consumption and how specific food choices are influenced by wider social, economic and lifestyle factors.

## Figures and Tables

**Table 1:** Reported intake of basic food (kg/person/year) by adult (19 years and above) and younger Australians (2 to 18 years) in 1995 and 2011. ^1^

Basic Food	Reporting Unit	Adults		Younger Australians	
		1995	2011	1995	2011
Fruit	Raw, edible portion	127.6	126.5	154.2	137.6
Vegetables (incl. mushrooms)	Raw, edible portion	127.3	123.9	83.3	85.0
Edible seaweed	Dried	<0.01	0.03	<0.01	0.04
Meat	Raw, edible portion	71.5	81.2	50.7	57.8
Seafood	Raw, edible portion	10.5	14.4	5.1	5.1
Dairy products	Whole milk equivalent	175.6	187.4	209.4	192.0
Eggs	Whole egg without shell	9.4	11.0	7.8	8.0
Grains	Whole grain equivalent	65.4	66.7	62.1	64.3
Beans, peas and pulses	Dried	1.5	2.2	0.9	1.4
Nuts and seeds	Dried	2.1	3.4	1.3	1.2
Sugar	Refined	20.8	20.4	28.5	21.9
Cocoa	Dried bean equivalent	0.8	1.5	1.5	2.0
Vegetable oil (incl. margarine)	Refined oil	13.2	11.8	11.9	10.4
Butter and ghee	Butter	2.5	2.3	2.3	2.2
Beer	Beer	69.9	63.4		
Wine	Wine	21.3	30.7		

^1^ Based on the 2011 age class distribution. Basic foods are expressed as raw food equivalents (edible portion only), after cooking and processing factors were applied as necessary.

**Table 2:** Reported intake of fruit (kg/person/year) by adult (19 years and above) and younger Australians (2 to 18 years) in 1995 and 2011. ^1^

Basic Food	Reporting Unit	Adults		Younger Australians	
		1995	2011	1995	2011
Fruit, raw	Raw, edible portion	51.2	58.2	45.3	61.1
Fruit, cooked	Raw, edible portion	9.4	5.6	5.6	3.3
Fruit, dried or reconstituted	Raw, edible portion	8.4	9.0	6.3	5.3
Fruit, juice	Raw, edible portion	58.6	53.6	96.9	67.9

^1^ Based on the 2011 age class distribution. The different forms of fruit consumption have been converted to raw equivalents (edible portion only), after cooking and processing factors were applied as necessary.

**Table 3:** Reported intake of vegetables (kg/person/year) by adult (19 years and above) and younger Australians (2 to 18 years) in 1995 and 2011. ^1^

Basic Food	Reporting Unit	Adults		Younger Australians	
		1995	2011	1995	2011
Potato and other starchy roots	Raw, edible portion	35.5	25.5	33.7	21.8
Mushrooms	Raw, edible portion	3.2	3.7	1.7	2.3
All other vegetables	Raw, edible portion	88.6	94.8	47.8	60.9

^1^ Based on the 2011 age class distribution. The different forms of vegetable consumption have been converted to raw equivalents (edible portion only), after cooking and processing factors were applied as necessary.

**Table 4:** Reported intake of meat and seafood (kg/person/year) by adult (19 years and above) and younger Australians (2 to 18 years) in 1995 and 2011. ^1^

Basic Food	Reporting Unit	Adults		Younger Australians	
		1995	2011	1995	2011
Beef	Raw, edible portion	31.3	30.5	21.9	22.1
Lamb	Raw, edible portion	7.3	6.7	4.5	3.8
Pig meat	Raw, edible portion	12.1	12.7	8.7	9.9
Chicken meat	Raw, edible portion	19.3	28.9	14.4	20.0
Turkey meat	Raw, edible portion	0.5	0.6	0.1	0.2
Duck meat	Raw, edible portion	0.1	0.4	<0.01	0.3
Fish	Raw, edible portion	8.2	12.2	4.3	4.6
Other animals of the sea	Raw, edible portion	2.3	2.2	0.8	0.6

^1^ Based on the 2011 age class distribution. The different forms of meat and seafood consumption have been converted to raw equivalents (edible portion only), after cooking and processing factors were applied as necessary.

**Table 5:** Reported intake of selected dairy products (kg/person/year) by adult (19 years and above) and younger Australians (2 to 18 years) in 1995 and 2011. ^1^

Basic Food	Reporting Unit	Adults		Younger Australians	
		1995	2011	1995	2011
Milk, whole	Whole milk equivalent	68.8	59.9	111.2	80.8
Milk, reduced fat	Whole milk equivalent	33.4	39.9	23.1	22.6
Cheese	Whole milk equivalent	50.7	55.6	45.8	54.3
Cream	Whole milk equivalent	3.1	3.3	1.9	2.3
Yogurt	Whole milk equivalent	6.0	11.6	6.2	10.6

^1^ Based on the 2011 age class distribution. The different forms of dairy product consumption have been converted to whole milk equivalents, after processing factors were applied as necessary.

**Table 6:** Reported intake of selected grains (kg/person/year) by adult (19 years and above) and younger Australians (2 to 18 years) in 1995 and 2011. ^1^

Basic Food	Reporting Unit	Adults		Younger Australians	
		1995	2011	1995	2011
Wheat, brown	Whole grain equivalent	7.4	7.4	5.9	6.7
Wheat, white	Whole grain equivalent	43.7	41.7	43.5	44.9
Rice, brown	Whole grain equivalent	0.3	0.5	0.2	0.4
Rice, white	Whole grain equivalent	7.6	9.5	7.1	7.1
Barley	Whole grain equivalent	0.3	0.2	0.2	0.2
Maize	Whole grain equivalent	1.5	1.4	2.5	2.2
Rye	Whole grain equivalent	1.6	1.7	0.6	0.8
Oats	Whole grain equivalent	2.9	4.0	2.1	2.0

^1^ Based on the 2011 age class distribution. The different forms of grain consumption have been converted to whole grain equivalents, after processing factors were applied as necessary.

## Supplementary Materials

The following are available online at www.mdpi.com/2072-6643/5/2/40/s1, Table S1: Reported intake of basic foods (kg/person/year) by Australians in 1995; Table S2: Reported intake of basic foods (kg/person/year) by Australians in 2011.
