# Supplementary material for: Changes in Food Intake in Australia: Comparing the 1995 and 2011 National Nutrition Survey Results Disaggregated into Basic Foods
Source: Foods. 2016 May 25;5(2):40. doi: 10.3390/foods5020040 (PMC5302341; doi:10.3390/foods5020040)
Supplement: Supplementary file 1 [file foods-05-00040-s001.pdf]

Supplementary Material: Changes in Food Intake in Australia: Comparing the1995 and2011 National Nutrition Survey Results Disaggregated into Basic Foods

Bradley Ridoutt, Danielle Baird, Kathryn Bastiaans, Gilly Hendrie, Malcolm Riley, Peerasak Sanguansri, Julie Syrette and Manny Noakes

Table S1. Reported intake of basic foods (kg/person/year) by Australians in1995 *.

Basic Food	Reporting Unit	Males							
		**2–3**	**4–8**	**9–11**	**12–13**	**14–18**	**19–49**	**50–69**	**70+**
Fruit	Raw, edible portion	177.8	145.5	142.5	137.8	148.2	123.5	119.2	117.9
Vegetables (incl. mushrooms)	Raw, edible portion	44.6	59.8	79.5	103.4	129.6	132.5	137.0	117.9
Edible seaweed	Dried	<0.01	<0.01	<0.01	<0.01	<0.01	<0.01	<0.01	<0.01
Meat	Raw, edible portion	25.2	37.4	52.7	62.0	84.3	96.2	75.6	60.1
Seafood	Raw, edible portion	1.9	3.1	5.5	5.8	7.4	10.9	12.9	9.0
Dairy products	Whole milk equivalent	219.6	197.0	215.0	269.9	263.1	200.1	157.5	139.8
Eggs	Whole egg without shell	4.7	7.0	8.1	10.1	11.4	10.7	10.1	8.2
Grains	Whole grain equivalent	40.6	57.4	72.4	79.3	82.0	80.0	63.2	55.3
Beans, peas and pulses	Dried	1.3	0.8	0.7	1.1	1.3	1.6	1.4	2.0
Nuts and seeds	Dried	0.7	1.3	1.6	1.3	0.9	2.6	1.9	1.2
Sugar	Refined	18.3	25.1	31.4	37.5	41.4	27.1	19.0	18.2
Cocoa	Dried bean equivalent	1.2	1.1	1.4	1.9	1.8	1.0	0.5	0.3
Vegetable oil (incl. margarine)	Refined oil	6.8	10.7	13.2	14.4	16.4	15.3	13.1	12.1
Butter and ghee	Butter	1.1	2.0	2.4	3.2	2.9	2.7	2.8	2.8
Beer	Beer					31.0	137.7	121.5	68.5
Wine	Wine					4.9	16.4	29.9	18.3

Table S1. *Cont.*

Basic Food	Reporting Unit	Females							
		**2–3**	**4–8**	**9–11**	**12–13**	**14–18**	**19–49**	**50–69**	**70+**
Fruit	Raw, edible portion	144.5	165.4	129.7	180.9	169.9	135.2	131.1	130.7
Vegetables (incl. mushrooms)	Raw, edible portion	44.6	58.3	75.8	106.7	104.4	120.9	128.2	117.1
Edible seaweed	Dried	<0.01	<0.01	<0.01	<0.01	<0.01	<0.01	<0.01	<0.01
Meat	Raw, edible portion	22.4	33.6	42.4	61.9	62.8	61.1	53.4	45.9
Seafood	Raw, edible portion	2.1	4.6	4.2	6.0	7.5	9.5	10.9	8.7
Dairy products	Whole milk equivalent	202.7	162.5	186.9	199.7	198.2	174.7	169.0	151.0
Eggs	Whole egg without shell	4.9	6.4	8.1	7.9	7.6	9.2	7.8	8.0
Grains	Whole grain equivalent	38.2	48.6	57.3	62.0	67.3	63.3	52.8	49.5
Beans, peas and pulses	Dried	0.9	0.4	0.4	0.7	1.0	1.4	1.2	1.4
Nuts and seeds	Dried	1.0	1.5	1.2	0.9	1.9	2.3	1.8	0.8
Sugar	Refined	15.9	22.2	26.1	32.8	28.3	19.8	15.1	14.8
Cocoa	Dried bean equivalent	0.9	1.1	1.6	1.7	2.1	1.1	0.7	0.3
Vegetable oil (incl. margarine)	Refined oil	6.8	9.7	11.4	12.9	12.6	12.8	11.5	10.8
Butter and ghee	Butter	1.2	1.7	2.4	3.2	2.4	2.2	2.1	2.6
Beer	Beer					10.8	18.6	14.4	4.5
Wine	Wine					2.4	24.9	21.3	11.7

* Basic foods are expressed as raw food equivalents (edible portion only), with cooking and processing factors applied where necessary.

Table S2. Reported intake of basic foods (kg/person/year) by Australians in 2011 *.

Basic Food	Reporting Unit	Males							
		**2–3**	**4–8**	**9–11**	**12–13**	**14–18**	**19–49**	**50–69**	**70+**
Fruit	Raw, edible portion	140.5	133.0	134.6	153.1	147.2	129.4	136.5	144.3
Vegetables (incl. mushrooms)	Raw, edible portion	47.0	72.3	89.3	119.1	124.5	132.7	132.3	125.6
Edible seaweed	Dried	<0.01	0.01	<0.01	<0.01	0.03	0.05	0.02	<0.01
Meat	Raw, edible portion	32.5	44.1	55.7	84.0	93.6	100.3	94.3	78.6
Seafood	Raw, edible portion	3.8	3.9	6.5	4.3	5.8	15.1	16.4	14.5
Dairy products	Whole milk equivalent	184.0	180.0	182.9	242.9	224.1	211.5	182.0	168.7
Eggs	Whole egg without shell	3.6	7.5	7.1	8.7	11.6	11.7	12.6	13.2
Grains	Whole grain equivalent	41.2	59.1	70.2	84.7	90.1	77.4	73.9	70.8
Beans, peas and pulses	Dried	1.9	0.9	1.1	0.9	2.1	2.2	1.7	1.9
Nuts and seeds	Dried	0.7	1.2	1.5	1.1	1.7	3.5	4.5	2.5
Sugar	Refined	12.2	19.2	22.2	29.9	31.0	25.3	20.5	20.9
Cocoa	Dried bean equivalent	0.9	1.4	2.1	3.2	2.1	1.4	1.5	1.4
Vegetable oil (incl. margarine)	Refined oil	6.0	9.3	11.3	13.7	13.2	13.0	12.1	12.3
Butter and ghee	Butter	0.9	2.1	2.6	3.1	2.8	2.2	3.0	3.6
Beer	Beer					15.3	115.7	134.5	76.8
Wine	Wine					0.6	16.9	50.0	35.5
**Basic Food**	**Reporting Unit**	**Females**							
		**2–3**	**4–8**	**9–11**	**12–13**	**14–18**	**19–49**	**50–69**	**70+**
Fruit	Raw, edible portion	111.0	126.8	133.6	138.9	149.8	114.8	123.4	137.0
Vegetables (incl. mushrooms)	Raw, edible portion	46.1	54.1	80.1	88.6	103.6	116.5	118.5	110.1
Edible seaweed	Dried	<0.01	0.03	0.2	<0.01	0.04	0.02	<0.01	<0.01
Meat	Raw, edible portion	29.7	34.1	50.8	63.7	71.6	67.1	66.2	64.9
Seafood	Raw, edible portion	2.4	5.0	3.4	8.0	7.0	11.6	16.8	14.6
Dairy products	Whole milk equivalent	192.9	148.3	181.1	219.9	196.0	177.3	179.9	165.5
Eggs	Whole egg without shell	4.9	5.9	8.8	9.2	9.5	9.9	10.0	10.5
Grains	Whole grain equivalent	41.4	49.8	57.6	67.6	66.7	59.6	56.7	52.9
Beans, peas and pulses	Dried	0.6	0.8	1.6	0.8	2.4	2.2	2.8	1.4
Nuts and seeds	Dried	0.9	0.6	0.9	1.2	1.4	2.8	4.0	2.4
Sugar	Refined	12.1	16.0	20.4	25.3	25.1	18.7	15.5	16.3
Cocoa	Dried bean equivalent	1.0	1.4	2.1	2.4	3.0	1.7	1.6	1.1
Vegetable oil (incl. margarine)	Refined oil	6.0	7.5	10.5	12.2	12.5	11.2	10.8	10.2
Butter and ghee	Butter	0.9	1.4	2.5	3.4	2.6	2.0	1.9	2.0
Beer	Beer					<0.01	12.6	10.2	8.0
Wine	Wine					0.1	25.9	49.2	28.5

* Basic foods are expressed as raw food equivalents (edible portion only), with cooking and processing factors applied where necessary.
